# An investigation on factors associated with malnutrition among underfive children in Nakaseke and Nakasongola districts, Uganda

**DOI:** 10.1186/s12887-015-0448-y

**Published:** 2015-09-24

**Authors:** Gilbert Habaasa

**Affiliations:** Population and Development Consult Limited, Kampala, Uganda

**Keywords:** Breastfeeding, Pastoralism, Childcare, Strategies

## Abstract

**Background:**

Malnutrition is one of the major causes of mortality and morbidity among under-five children in Sub Saharan Africa. To understand the factors associated with malnutrition among under-five children, a study was conducted in Nakaseke and Nakasongola districts of Uganda.

**Method:**

Cross sectional secondary data of 104 underfive children in Nakaseke and Nakasongola districts was used. Epi Info programme-Nutrition module and Stata statistical softwares were used in analyses. Descriptive statistics, cross tabulations and binary logistic regression results were generated.

**Result:**

Stunting was found to be the most malnutrition condition with the highest prevalence (38.5 %) in the two districts followed by wasting (16.5 %) and underweight (13.5 %) respectively. Results also showed that children aged 39–59 months were less likely to be underweight than those aged below twelve months. Children of peasant farmers were more likely to be stunted than their counterparts with mothers in pastoralist’s family. No significant factors were found to be associated with wasting among the underfive children in the two districts although the prevalence was slightly higher than that of child underweight.

**Conclusion:**

The study is essential in pointing out the particular age-groups among underfive children as well as the maternal occupations that may be factors associated with malnutrition in the districts of Nakaseke and Nakasongola. The author recommends exclusive breast feeding and proper complementary feeding especially among children under three years. Furthermore, special arrangement could be put in place to have children of mothers engaged in cultivation brought to them regularly for breastfeeding.

## Background

The World Health Organization in a recent report estimates that there are 178 million malnourished children across the globe, and at any given moment, 20 million of these are suffering from the most severe form of malnutrition. Malnutrition contributes between 3.5 and 5 million annual deaths among under-five children. UNICEF estimates about 195 million children suffering from malnutrition across the globe [1]. This consequently affects the intelligence level of children, their behaviour and school performance. The impaired mental development is taken as the most serious long-term handicap associated with underfive malnutrition. In Sub-Saharan Africa, 41 % of under-five children are malnourished and deaths from malnutrition are increasing on daily basis in the region [2].

In Uganda, malnutrition remains a serious health and welfare problem affecting the under-five children to whom it contributes significantly to mortality and morbidity. According to Uganda Demographic and Health Survey of 2011, four in ten Ugandan children under-five years of age (33 %) are stunted (short for their age), six percent are wasted (thin for their height), and 14 percent are underweight (low weight for age) [3]. Indeed, these statistics may not be different from the districts of Nakaseke to Nakasongola in Uganda. Worthy to note is that the Ugandan government has put in place a number of initiatives aimed at reducing the prevalence of malnutrition in the country; The 2004/2005 Uganda food and nutrition policy reform, the Uganda Vision 2040 and 2010–2015 National Development Plan [4, 5].

Little improvement on underfive nutrition indicators in Uganda has been realized for the past 15 years [3]. Given the number of studies on malnutrition among underfive children in developing countries, there is need to examine if similar factors are associated with malnutrition among underfive children in Uganda particularly in the districts of Nakaseke and Nakasongola.

The specific study objectives were; (i) To ascertain the prevalence of malnutrition among underfive children, establish the relationship (ii) between demographic factors and malnutrition, (iii) between socio-economic factors and malnutrition among underfive children. The hypotheses tested were (i) There is no relationship between demographic factors and malnutrition among under-five children. (ii) There is no relationship between socio-economic factors and malnutrition among underfive children.

## Methods

Cross sectional data of 104 underfive children (full sample) was obtained from Africa Innovations Institute (AfrII). Categorical data was collected in 2012 from households within the districts of Nakaseke and Nakasongola. The two districts were covered by the AfrII project “Adaptation to the impact of climatic variability on food and health security in the cattle corridor of Uganda” funded by International Development Research Centre (IDRC), Canada. Five public health experts from Makerere University School of public health were engaged in taking of anthropometry measurements for the underfive children. The full methodology and sampling procedures undertaken during data collection can be found elsewhere [6].

Child variables that included age, sex, height and weight were entered in Epi Info7 software-nutrition module to generate measurement indices of height-for-age, weight-for-age and weight-for-height. The indices generated were compared with standard reference values for CDC 2000 growth references to obtain the Z-scores. For this study, three indices of malnutrition that included stunting, underweight and wasting were determined among all the under-five children. Children whose height-for-age Z-score was below minus 2 standard deviation from the median of the reference population were classified as stunted. Children having weight-for-age Z-score less than minus 2 standard deviation from the median of the reference population were regarded as underweight. Similarly, all the children under-five years whose weight for height Z-scores were less than minus 2 standard deviation were regarded as wasted [7].

The nutrition indicators of stunting, wasting and underweight were entered in stata programme and then merged with the demographic and socio-economic data for analysis. Descriptive statistics as well as cross tabulations were generated. Cross tabulations with Pearson Chi Square (${\chi}^{\mathtt{2}}$) tests were performed to establish the association between under-five malnutrition with demographic and socio-economic characteristics of the children. At multivariate analysis, a binary logistic regression model was fitted to ascertain the factors associated with malnutrition among children underfive years. The independent variables entered included; sex of child, age of child, birth order, birth interval, mother’s age at birth, mother’s education level, mother’s marital status and mothers occupation. The dependent variables were stunting, underweight and wasting.

### Ethics

Clearance was obtained from the Ethical board of Uganda National Council for Science and Technology to conduct the study. A written and signed informed consent was obtained from parents and guardians for the participation of their children in the study. Respondents were assured that the information would be used for intervention and academic purposes only. There were no blood samples taken from the respondents and the study did not pose any danger to them. The data collected did not have personal information like names and besides the variables were coded for confidentiality purpose.

The author also obtained the dataset and official permission from Africa Innovations Institute to undertake this study.

## Results

### Background characteristics

The background characteristics are divided into demographic and socio-economic factors as presented below.

### Demographic factors

More than half of the under-five children in the study were females and majority were aged 37–59 months. Half of the children were of birth order 1–2 with a few in the birth order of 3–4 and 5+ order respectively. Most of the children were of birth intervals equal or less than two years. On the age of the mother at birth, majority of the children had their mothers aged 30–39 years while quite a significant proportion was also from children whose mothers at birth were aged 20–29 years. Few of the children were from mothers aged less than 20 years and 40–49 years at birth respectively. The details of the findings on the demographic factors among underfive children are presented in Table 1.

**Table 1:** Demographic factors of the underfive children

Demographic factors (*n* = 104)	Frequency	Percentage (%)
Sex of Child		
Male	51	49
Female	53	51
Age of the child (months)		
≤ 12	76	73.1
13–36	16	15.4
37–59	12	11.5
Birth Order		
1–2	52	50
3–4	26	25
5+	26	25
Birth Interval (years)		
≤ 2	46	44.2
3–4	43	41.3
5–6	15	14.5
Age of mother at birth (years)		
<20	16	15.4
20–29	34	32.7
30–39	42	40.4
40–49	12	11.5

### Socio-economic factors

Most of the mothers had received primary level education and quite a few had never been to school. Majority of the mothers were married or cohabiting. Most of the mothers were peasant farmers followed by those engaged in business, public service and pastoralism. Details are presented in Table 2.

**Table 2:** Socio-economic factors of the underfive children

Socio-economic factors (*n* = 104)	Frequency	Percentage (%)
Mothers’ educational level		
No education	16	15.4
Primary	76	73.1
Secondary+	12	11.5
Marital status of the mother		
Never Married/Separated	35	33.7
Married/Cohabiting	69	66.3
Mothers’ Occupation		
Peasant farmer	52	50.0
Pastoralist	14	13.4
Business/civil servant	32	30.8
Handcrafts	6	5.8

### Levels of malnutrition among underfive children

Results indicate that stunting was the most common malnutrition problem among underfive children in Nakaseke and Nakasongola district. There was also quite a high prevalence of wasting and underweight among underfive children given the fact that the sample size of children was not very big. On the levels of malnutrition by district; results indicate that Nakaseke district had a higher prevalence of stunting than Nakasongola. Similarly, Nakaseke had higher prevalence of child wasting and underweight respectively than Nakasongola district. The levels of malnutrition among children underfive years in Nakaseke and Nakasongola districts in Central Uganda are presented in Table 3.

**Table 3:** Levels of malnutrition among underfive children

Malnutrition Index	Overall status (%)	Nakaseke (*n* = 54)	Nakasongola (*n* = 50)
Stunting	38.5	23 (42.6 %)	17 (34.0 %)
Wasting	16.5	12 (22.2 %)	5 (10.2 %)
Underweight	13.5	9 (16.7)	5 (10.0 %)

### Relationship between demographic and socio-economic factors with malnutrition

The study showed that slightly more females were stunted compared to the males. For wasting and underweight, xfemales were equally more wasted and underweight respectively than their male counterparts despite the fact that there was no significant relationship between sex of child and malnutrition.

On the age of a child, there was a significant relationship between age of child and underweight (*p* = 0.041**< 0.05). There were few underweight children aged 13–59 months unlike those aged 12 months and below.

For birth order, stunting was highest among children of birth order 1–4 than those of order 5 and above. Children of birth order 3–4 were more wasted than those of birth order 1–2 or 5+. Similarly, underweight was highest among children of birth order 3–4.

On the birth interval, stunting was highest among underfive children with birth interval of 3–4 years.

Results also indicate that there were more stunted children among mothers aged 30–39 years than those aged 20–29 years or even 40–49 years. There was however no significant relationship between age of mother at birth and stunting. However, there were more wasted children among mothers aged 20–29 years unlike other age groups. It is indicated that majority of underweight children were from mothers aged 40–49 years. There was no significant relationship between age of mother and malnutrition among underfive children.

For wasting, however, more children of birth interval ≤ 2 years were wasted. On underweight, only few cases of children with birth interval 4 years and below were underweight. There was however no significant relationship between birth interval and all the malnutrition indices that is stunting, wasting and underweight.

There was no significant relationship between mother’s education level and malnutrition indicators. The relationships of mother’s education with stunting, wasting and underweight were statistically insignificant since the p-values were greater than 0.05 at 95 % confidence interval.

On the marital status, majority of the stunted children were from mothers who were married or cohabiting. Similarly, there were more wasted and underweight children among married or cohabiting couples. There was however no significant relationship between marital status and malnutrition.

There was a significant relationship between mothers occupation and underfive child stunting (*p* = 0.05). More stunted children were from peasant farmers. In the same vein, wasting and underweight was common among peasant farmers and pastoralists. Results on the relationship between demographic and socio-economic factors with malnutrition indicators among underfive children are presented in Table 4.

**Table 4:** Bivariate associations between demographic and socio-economic factors with malnutrition among underfive children

Explanatory variable	Total children	Stunting			Wasting			Underweight		
		Stunted (%)	${\chi}^{\mathtt{2}}$	*p*	Wasted (%)	${\chi}^{\mathtt{2}}$	*p*	Underweight (%)	${\chi}^{\mathtt{2}}$	*p*
Sex of the child			0.011	0.546		3.302	0.069		1.839	0.159
Male	39	15 (38.5)			3 (7.7)			2 (5.1)		
Female	48	19(39.6)			11 (22.9)			7 (14.6)		
Age of child (months)			1.992	0.737		0.788	0.94		9.952	0.041*
≤ 12	76	32 (42.1)			13 (17.1)			9 (11.8)		
13–36	16	4 (25)			2 (12.5)			0		
37–59	12	6 (50)			2 (16.7)			5 (41.7)		
Birth Order			0.438	0.803		2.921	0.232		5.378	0.068
1–2	43	19 (44.2)			3 (6.9)			3 (6.9)		
3–4	18	8 (44.4)			4 (22.2)			4 (22.2)		
5+	17	6 (35.3)			3 (17.6)			0		
Birth Interval (years)			5.085	0.079		0.153	0.926		0.795	0.672
≤ 2	38	15 (39.5)			6 (15.7)			4 (10.5)		
3–4	17	11 (64.7)			2 (11.8)			2 (11.8)		
5–6	6	1 (16.7)			1 (16.7)			0		
Age of mother at birth (years)			3.135	0.371		1.351	0.717		1.503	0.681
< 20 Years	16	6 (37.5)			2 (12.5)			2 (12.5)		
20–29 Years	41	15 (36.6)			7 (17.1)			3 (7.3)		
30–39 Years	23	13 (56.5)			3 (13.0)			3 (13.0)		
40–49 Years	7	2 (28.6)			0			0		
Mother’s education level			1.135	0.769		3.525	0.317		2.866	0.413
No education	11	3 (27.3)			3 (27.3)			2 (18.2)		
Primary	57	25 (43.9)			6 (10.5)			4 (7.0)		
Secondary+	11	5 (45.6)			2 (18.2)			0		
Marital Status			1.331	0.275		1.55	0.362		0.032	0.584
Never married/Separated	34	11 (32.4)			3 (8.8)			3 (8.8)		
Married/Cohabiting	56	25 (44.6)			11 (19.6)			6 (10.7)		
Mothers’ occupation			11.03	0.05*		3.101	0.684		3.419	0.636
Peasant farmer	44	20 (45.5)			6 (13.6)			5 (11.4)		
Pastoralist	11	1 (9.1)			2 (18.2)			0		
Business/civil servant	15	9 (60)			3 (20)			0		
Handcraft	6	2 (27.3)			0			0		

*Statistically significant at 95 % Confidence Interval

### Factors associated with malnutrition among underfive children

Results in Table 5 indicate that children aged 37–59 months were less likely to be underweight (OR = 0.76) than their counterparts who were aged 12 months and below (reference category) in Nakaseke and Nakasongola districts. In fact children aged 37–59 months and child underweight were statistically significant (*p* = 0.03**< 0.05) at 95 % confidence interval.

**Table 5:** Factors associated with malnutrition among underfive children in Nakaseke and Nakasongola districts

Variable	Stunting			Wasting			Underweight		
	OR	*β*	*p*	OR	*β*	*p*	OR	*β*	*p*
Sex of the child (Male^a^)									
Female	1.07	0.48	0.89	3.36	2.34	0.08	2.98	2.48	0.19
Age of child (≤ 12^a^ months)									
13–36 months	0.38	0.26	0.16	0.79	0.65	0.78	0.67	0.59	0.65
37–59 months	0.76	0.46	0.66	0.79	0.65	0.78	4.14	2.74	0.03*
Birth Order (1–2^a^)									
3–4	0.74	0.42	0.59	3.62	2.98	0.12	3.62	2.99	0.12
5+	0.69	0.42	0.55	2.92	2.56	0.22	4.40	5.78	0.26
Birth Interval (≤ 2^a^ years)									
3–4 years	2.33	1.44	0.17	0.71	0.63	0.70	1.14	1.05	0.88
5–6 years	1.86	2.18	0.59	0.72	0.66	0.72	1.04	0.84	0.96
Age of mother at birth (< 20 Years^a^)									
20–29 Years	0.80	0.50	0.72	1.27	1.11	0.78	0.48	0.47	0.46
30–39 Years	1.45	0.99	0.58	0.90	0.88	0.92	0.89	0.88	0.91
40–49 Years	0.27	0.33	0.28	2.17	1.32	0.21	0.19	0.24	0.20
Mother’s education level (No education^a^)									
Primary	3.09	2.59	0.18	0.29	0.23	0.12	0.31	0.29	0.22
Secondary+	4.00	4.24	0.19	0.78	0.83	0.81	0.40	0.39	0.36
Marital Status of the mother (Never married/Separated^a^)									
Married/Cohabiting	2.04	0.98	0.14	2.33	1.62	0.22	1.14	0.85	0.86
Mothers’ occupation (Peasant^a^)									
Pastoralist	0.12	0.13	0.05*	2.53	2.39	0.33	2.11	2.61	0.55
Business/civil servant	2.71	1.82	0.14	1.58	1.24	0.56	1.15	1.39	0.91
Handcraft	1.23	1.74	0.92	0.24	0.27	0.21	1.15	1.38	0.90

*Statistically significant at 95 % Confidence Interval

^a^Reference category

Findings also indicate that there is a significant relationship between woman’s occupation and stunting among underfive children (*p* = 0.05) in Nakaseke and Nakasongola districts. Children whose mothers were pastoralists (OR = 0.12) were less likely to be stunted unlike their counterparts whose mothers were peasant farmers (reference category) as shown in Table 5.

## Discussions

### Conceptual frame work

**Fig. 1:**
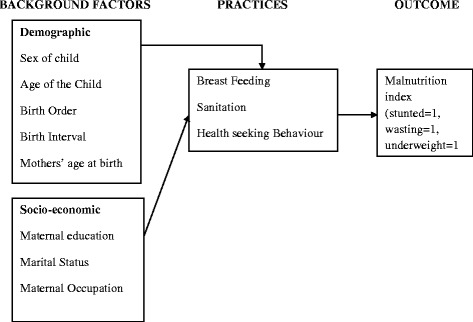
Conceptual framework showing factors associated with malnutrition among underfive children. Source: Modified and adopted UNICEF conceptual framework on nutrition, 1998

Figure 1 shows that in developing countries and particularly in Sub-Saharan Africa, under-five child malnutrition is normally determined by a large number of factors to the extent that it sometimes becomes difficult to predict the risk factors [8]. Such factors act through a number of interrelated proximate determinants to bring about underfive malnutrition that is stunting, underweight and wasting. The demographic and socio-economic factors such as age of child, birth order, mothers age at birth, mothers education level, marital status as well as maternal occupation work through proximate variables like the duration of breast feeding, sanitation and mothers health seeking behaviours to influence underfive malnutrition. With some modification, the UNICEF conceptual framework on nutrition strategy and child survival was adopted for this study [9]. Some of the variables in the UNICEF conceptual framework were not applicable to the study design adopted hence were not adopted in the paper for instance inadequate health facilities.

Study findings indicated a significant relationship between age of child and malnutrition. Children aged 37–59 months were found less likely to be underweight (OR = 0.76) than their counterparts who were aged 12 months and below in the districts of Nakaseke and Nakasongola. Similar findings have been reported at national level where the proportion of underweight children is lowest among children 36–59 months old and highest among those 6–8 months old [3]. Other studies in Vietnam, India, Nigeria and Kenya have reported similar findings [10–12]. Perhaps, this could be associated with the fact that when children are weaned especially after exclusive breastfeeding in the first six months, some women go back to their work places and devote less time to the care of their children. The findings are however contrary to the study in Ethiopia that found out that underweight had a positive linear relationship with age of a child [13].

Furthermore, maternal occupation emerged as a significant factor that could be associated with underfive stunting in the same districts. Children from mothers who were pastoralists were found 0.12 times less likely to be stunted than children whose mothers were peasant farmers. Mothers engaged in pastoralism are believed to supplement the nutrition value of their children with cow milk and other milk products which consequently reduces the risk of stunting unlike the peasant farmers. According to a study in Botswana [14], crop cultivators were more likely to have stunted children. Similarly, a study done in Vietnam found out that children from mothers who were crop cultivators had an increased risk of stunting because they rarely get time to care for their children hence end up leaving them under the care of elder siblings or inexperienced maids [10]. In another study, by Olwedo, Mworozi and Bachou found some mothers particularly peasant farmers in most cases failed to provide complementary feeding to their children because they could not afford [15]. This factor therefore proposes that the economic engagement of the mother that particularly promote extended separation of the child from the primary caregiver may be detrimental consequently resulting into malnutrition especially stunting. However, observations from other studies suggest that women engagement in crop cultivation compared to informal businesses may be a reflective of the better resources and childcare practices within households particularly those with suitable childcare [14, 16, 17]. Worthy to note is that the above studies conducted elsewhere do not specifically compare children from peasant farmers with those from pastoralism communities on the stunting condition.

The present study reports on the level of malnutrition as well as the factors that could be associated with malnutrition among underfive children in Nakaseke and Nakasongola districts. Given that a substantial number of 104 underfive children were studied, the study may be regarded as a reasonable reflection of the nutritional status of underfive children in the above districts. Worthy to note also is that the study reveals that malnutrition is a problem that affects 38.5 % (stunting), 16.5 % (wasting) and 13.5 % (underweight) of children underfive years in the two districts of Nakaseke and Nakasongola. The findings are slightly higher than the Uganda national figures of stunting at 33 %, and wasting at five percent. There is an almost similar proportion of underweight children with the national prevalence of 14 % according to the 2011 Uganda Demographic and Health Survey [3]. Among the study limitations, the dataset used missed out some variables of interest on child malnutrition that included duration of breast feeding and Body Mass Index of the mother. The sample size of 104 respondents was also relatively small hence could have had an effect on the outcomes of the study.

### Recommendations

The study recommends exclusive breast feeding and proper complementary feeding especially among children under three years. In line with UNICEF and WHO recommendations, there is need for exclusive breast feeding during the first six months of life and thereafter semi-solid complementary foods are introduced up to at least two years or more. This will consequently reduce on the underweight children who are mostly aged less than three years in the districts of Nakaseke and Nakasongola.

The study also recommends a special arrangement for mothers engaged in cultivation to have their children breastfed regularly by having their babies brought to them in the gardens at regular intervals. The mothers could also visit their babies at home regularly from their gardens to ensure that proper nutrition is given to their children. This may contribute to a reduction in stunting especially among children of peasant farmers who were found to have increased levels of malnutrition than the rest of the children with mothers of other occupations.

There is need for a bigger study to be carried out in the districts of Nakaseke and Nakasongola covering more children to establish the factors associated with underfive malnutrition. Perhaps another study may establish significant factors like education of mother, sex of child, birth order, birth interval, age of mother and marital status since most of them were found significant in the literature review.

## Conclusion

Results from the analyses confirm that age of a child and maternal occupation could be some of the factors associated with malnutrition among underfive children in Nakaseke and Nakasongola district. The study therefore underscores the age groups that could be prone to malnutrition challenges as well as the particular occupations among women that could pose a risk of malnutrition to the underfive children. This then could give a focus to policymakers in the designing of strategies aimed at combating malnutrition among children below five years. It should be noted however that some other factors could be associated with malnutrition among the underfive children and not necessarily the age of a child or even mothers occupation for instance, unemployed mother who breastfeeds her child could have a well fed child. Similarly, on the age of a child, it could be that the majority of the underweight children were just under a specific age group and not necessarily associated with malnutrition condition.
